# Thermomechanical Tailoring of a DLP-Printable Shape Memory Polyurethane for Vascular Graft Applications

**DOI:** 10.3390/ma19091862

**Published:** 2026-05-01

**Authors:** Ozan Azğüler, Mihrigül Ekşi Altan

**Affiliations:** Department of Mechanical Engineering, Yıldız Technical University, Istanbul 34349, Türkiye

**Keywords:** 4D printing, resin formulation, photo-polymerization, DLP, shape-memory, vascular graft

## Abstract

The increasing prevalence of cardiovascular diseases highlights the need to develop vascular grafts that match the mechanics of native vascular tissue and offer functional adaptability. This study reports the development and systematic optimization of a shape-memory polyurethane acrylate (PUA)-based photocurable resin for digital light processing (DLP)-based four-dimensional printing (4DP) applications. Resin formulations were designed by controlling hard/soft segment ratios, reactive diluent content, and crosslink density to position the glass transition temperature (T_g_) within the physiological range (25–40 °C). Thermomechanical characterization was performed via dynamic mechanical analysis (DMA) and tensile testing, while a full-factorial Design of Experiments (DoE) approach was applied to optimize DLP process parameters—namely layer thickness, exposure time, and post-curing time. The developed resin formulation yielded a T_g_ of 38 °C as determined by DMA. Following process optimization, regression models showed high statistical fit (R^2^ > 99%), and experimental validation under optimal conditions (layer thickness: 82.83 µm, exposure time: 11 s, post-curing: 2 min) resulted in an elongation at break of 64.0 ± 3.4%, a Young’s modulus of 10.9 ± 0.1 MPa, and a tensile strength of 6.2 ± 0.3 MPa. The optimized system exhibited thermally triggerable shape memory behavior at near-body temperature, with mechanical properties consistent with natural arterial tissue benchmarks. These findings demonstrate a promising material design strategy for DLP-based 4D-printed vascular structures.

## 1. Introduction

Cardiovascular diseases account for approximately one-third of deaths worldwide and represent a significant global health problem [1,2]. In ischemic heart disease, peripheral artery disease, and other vascular pathologies, surgical interventions such as bypass or vascular replacement are required to restore blood flow [3]. In these applications, autografts such as the saphenous vein, internal mammary artery, and radial artery are considered the standard of care due to their low immunogenicity and high patency rates. However, they have various limitations, including donor site trauma, risk of infection, and pain [4,5,6,7]. Therefore, the development of artificial vessels that can mimic natural vessels in both morphology and mechanics is of great clinical importance [7,8].

Two fundamental requirements stand out in artificial vessel design: mechanical compatibility and biocompatibility [9]. Parameters such as the Young’s modulus, tensile strength, and elongation at break of natural vessels have been reported in detail in the literature [10,11,12,13,14]. Notably, Camasão and Mantovani reported that the circumferential Young’s modulus of human femoral arteries ranges from 9 to 12 MPa, and that elongation at break values of 63–76% are characteristic of arterial tissue under physiological loading conditions [9]. In contrast, commercially available polyethylene terephthalate (PET, Dacron^®^) and polytetrafluoroethylene (PTFE, Teflon^®^)-based grafts have significantly higher Young’s modulus compared to natural vascular tissues and can cause compliance mismatch, especially in small-diameter applications [15,16]. Wang et al. reported that this stiffness discrepancy between synthetic grafts and native vessels leads to disturbed hemodynamics and elevated shear stress at anastomosis sites, increasing the risk of intimal hyperplasia and graft failure over time [7]. Biodegradable polymers (polycaprolactone (PCL), poly-lactic acid (PLA), polyglycolic acid (PGA)) offer the advantage of controlled degradation but have limitations long-term mechanical stability [17,18,19,20]. Liu et al. demonstrated that PCL/PLLA-based electrospun scaffolds, while exhibiting adequate initial mechanical properties, undergo a significant reduction in stiffness over time due to hydrolytic degradation, thereby compromising their structural integrity under sustained hemodynamic loading [18]. Natural polymers (gelatin, collagen, silk fibroin, chitosan, etc.) are advantageous for biocompatibility but cannot always provide sufficient mechanical strength under physiological pressure [21,22,23,24]. For example, Niu and Galluzzi reported that hyaluronic acid/collagen nanofiber tubular scaffolds, despite their excellent cell compatibility and potential for endothelialization, exhibited mechanical properties insufficient to withstand arterial blood pressure without additional reinforcement [22]. Therefore, the development of new polymer systems with properties similar to natural vessel mechanics and adjustable characteristics presents an important materials engineering problem.

The methods used in artificial vessel production range from textile-based techniques to advanced manufacturing technologies [7]. In addition to methods such as weaving, knitting, and braiding, techniques such as electrospinning and thermally induced phase separation (TIPS) have also been reported in the literature [25,26,27,28]. However, there are limitations in pore control, mechanical strength, and structural reproducibility [8]. Three-dimensional printing (3DP) technologies, which have developed rapidly over the past twenty years, have gained significant momentum in biomedical applications by enabling the production of complex geometries and customized structures [29,30]. 4DP, an extension of this approach, refers to the production of structures that can change shape over time in response to external stimuli [31,32]. Zarek et al. demonstrated the feasibility of 4D-printed shape memory based endoluminal medical devices, showing that thermally responsive polymers can be programmed to deploy from a compact temporary configuration to a functional permanent geometry upon exposure to body temperature, thereby highlighting the clinical potential of this strategy for minimally invasive implantation [8].

One of the most extensively researched classes of materials in 4DP applications is the shape memory polymer (SMP) group [33,34]. In these materials, the shape memory effect depends on the morphological balance between the permanent network structure of rigid segments and the transient phase of soft segments [35]. The shape memory cycle, which is triggered by heat, consists of the following steps: heating the sample above its T_g_, deforming it into a temporary shape, cooling it below T_g_, and then reheating it above T_g_ to return it to its permanent shape [36,37]. In biomedical applications, various SMP materials, including polyurethane (PU), PCL, PLA, PGA, and hydrogel systems, have been investigated [38,39]. Dayyoub et al. reviewed the current state of shape memory polymers as smart materials and emphasized that the precise control of T_g_ is the most critical design parameter for biomedical applications, as it directly governs the temperature at which the shape memory effect is activated [37]. However, in vascular applications, precise adjustment of T_g_ over the range from room temperature to body temperature is a critical requirement for minimally invasive implantation [40].

Recent studies have demonstrated the potential of shape memory polymers such as poly(d,l-lactide-co-trimethylene carbonate) (PLMC) for biomedical 4D printing applications, particularly due to their biocompatibility and ability to recover near physiological temperatures. For instance, Wan et al. fabricated PLMC-based shape-changing structures via direct ink writing and demonstrated thermally triggered shape recovery at temperatures close to physiological conditions, confirming the viability of this material class for implantable smart devices [41]. However, direct thermal stimulation of PLMC presents practical limitations for in vivo applications, as the poor thermal conductivity of polymers necessitates heating the entire surrounding tissue, leading to slow, non-uniform actuation and a potential risk of thermal damage [42]. To overcome this, athermal actuation strategies based on functional nanofillers have been explored, in which remote stimuli generate heat locally within the polymer matrix. Choudhury et al. incorporated Fe_3_O_4_ nanoparticles into PLMC to enable remote shape recovery under an alternating magnetic field at near-physiological temperatures, thereby demonstrating the superiority of this athermal approach over direct heating for in vivo deployment [42]. Building on this concept, near-infrared (NIR) light has emerged as an even more precise stimulus, enabling spatially and temporally controlled actuation. In NIR-responsive PLMC–polydopamine composites, photothermal conversion occurs locally within the matrix, raising the temperature above T_g_ before significant heat dissipation to surrounding tissues, which underlies the rapid and spontaneous shape recovery observed under low-power irradiation [43]. Compared with these PLMC-based systems that use nanofillers or external field-generating equipment, the present study explores DLP-processable polyurethane acrylate resins as a complementary approach, leveraging the advantages of photo-crosslinking to achieve high processing resolution and a tunable network architecture for potential biomedical vascular graft applications.

Vat polymerization (VP) methods, particularly stereolithography (SLA) and digital light processing (DLP), provide a suitable infrastructure for biomedical-scale structures due to their high resolution and surface quality. The DLP method shortens production time by curing the entire layer at once using a digital micromirror device (DMD) [44,45,46]. Bucciarelli et al. reported that DLP-based vat photopolymerization enables the fabrication of high-aspect-ratio microstructures with sub-100 µm dimensional accuracy, which is particularly relevant for producing hollow vascular geometries with controlled wall thickness [47]. However, the number of resin systems suitable for DLP that are both mechanically tunable and exhibit shape memory properties is limited [48]. While polyurethanes offer advantages in elasticity and biocompatibility, they are not directly photopolymerizable; in contrast, PUA systems offer a promising alternative by combining elastomeric behavior with photopolymerization [49]. Deng et al. demonstrated that urethane acrylate-based photosensitive resins can be formulated to achieve elastomeric mechanical behavior in DLP-printed constructs, and that the hard/soft segment ratio is a key determinant of both T_g_ and elongation at break [50].

The ISO 7198:2016 standard defines criteria for the mechanical characterization of vascular grafts and recommends evaluating parameters such as Young’s modulus, tensile strength, and elongation at break [9,51]. The development of a DLP-compatible polymer system that can approximate natural vessel mechanics and exhibit thermally triggered shape memory behavior requires a holistic approach to material chemistry and network architecture.

In this study, the thermomechanical adaptation and material optimization of a DLP-processable shape-memory PUA-based resin system were systematically investigated. PUA-based photocurable resin formulations were systematically designed; the objectives were to adjust the T_g_ to the 25–50 °C range, achieve mechanical properties comparable to those of natural vascular tissues, and ensure compliance with DLP requirements. This property offers a significant functional advantage for minimally invasive implantation strategies. In the context of 4D printing (4DP), shape recovery behavior of the fabricated constructs enables programmable structural transformation upon physiological stimulation, bridging the gap between static implant design and dynamic tissue function. Thus, DLP-based 4DP emerges as a promising manufacturing pathway for the next generation of smart vascular implants that can adapt their geometry in response to body temperature. This property offers a significant functional advantage for minimally invasive implantation strategies. In conclusion, the process–structure–property relationship between the resin composition and the thermomechanical response has been established, and a controlled material design approach for vascular structures produced by 4DP has been developed. This study contributes to the engineering-based optimization of DLP-compatible shape memory polymer systems for vascular applications.

## 2. Materials and Methods

### 2.1. Materials

In this study, various monomers, oligomers, crosslinkers, and photo-initiators were used in the resin formulation. An aliphatic urethane acrylate oligomer (UM-2200C1) was used as the soft segment and a urethane dimethacrylate oligomer (UDMA-UM3200) as the hard segment to control the network architecture in the resin system. Poly(ethylene glycol) dimethacrylate (PEG600DMA) was added to increase chain mobility and promote elastomeric behavior. Triethylene glycol dimethacrylate (TEGDMA) was added as a reactive diluent to reduce viscosity and improve processability. Lauryl acrylate (LA) was selected to increase system flexibility and lower the T_g_. Additionally, isobornyl acrylate (IBOA) was used as both a reactive diluent and a mechanical modifier, as it supports mechanical strength while reducing resin viscosity to facilitate processing. Trimethylolpropane triacrylate (TMPTA) was used as a crosslinker to strengthen the network structure due to its triple-functional structure. In the photopolymerization process, the phosphine oxide-based photo-initiator Darocur-TPO, which exhibits strong absorption at a wavelength of 405 nm, was used in combination with the α-hydroxybenzophenone derivative Darocur-1173. TPO provides deep curing and high radical production yield, while Darocur-1173 supports surface curing, contributing to the formation of a homogeneous network. UM-2200C1, UDMA-UM3200, PEG600DMA, TEGDMA, and TMPTA materials were supplied by MCT Chem Co. (Istanbul, Turkiye), the IBOA was supplied by RAHN A.G. (Istanbul, Turkiye) and the Darocur materials were supplied by Sigma Aldrich (Sigma Aldrich, St. Louis, MO, USA).

### 2.2. Resin Preparation

Various resin formulations were prepared by combining soft and hard oligomers in different ratios, reactive diluents, and additives. The starting composition for the formulation studies was determined by calculating the theoretical T_g_ values using the Fox equation. A T_g_ value measured by DMA in the range of 25–50 °C will ensure the applicability of the shape memory property in in vivo applications such as vascular function. Therefore, the initial approach aimed to achieve a T_g_ range of 25–50 °C for the formulation developed. A PUA-based study was referenced, and the initial formulation was determined by evaluating flow, viscosity, and printability [49]. The formulation presented in Table 1 is PUA-based and contains two types of PUA resin, the reactive diluent TEGDMA, the crosslinker TMPTA, and a photo-initiator composition comprising TPO and 1173.

**Figure 1:**
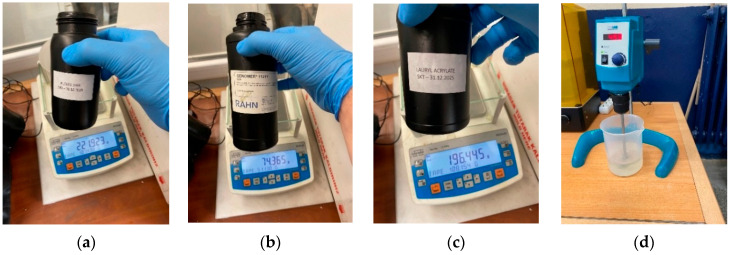
Preparation of resin formula: (**a**–**c**) Different raw materials weighing; (**d**) Mixing process.

During resin mixing, all components were weighed according to the target weight ratios for each formulation, placed in amber-colored glass bottles, and mechanically stirred at 1000–1500 rpm at room temperature for 60–90 min, until a homogeneous solution was obtained. The mixtures were allowed to settle for at least one day to remove trapped air bubbles. The prepared resins were stored in light- and air-protected containers at room temperature prior to DLP-based 3DP experiments. Images of raw material weighing (a–c) and mixing (d) are shown in Figure 1 to illustrate the resin preparation protocol and support the reproducibility of the formulation procedure.

### 2.3. DLP Printing Parameters and Design of Experiments (DoE) Methodology

All samples were printed at room temperature (23 ± 2 °C) using an Anycubic Photon M3 Plus DLP 3D printer (Shenzhen Anycubic Technology Co., Ltd., Shenzhen, China) equipped with a 405 nm LED light source. For the initial samples, where the primary goal was to achieve the T_g_ value, a layer thickness of 50 µm was selected. To ensure adequate adhesion to the print platform, the first two layers were cured for 25 s, and the subsequent layers for 7 s. The exposure parameters were adjusted to maintain dimensional accuracy while ensuring sufficient cure depth. After printing, the samples were carefully removed from the platform and washed in an ethanol bath for 2 min to remove uncured residues. To ensure complete cross-linking of the samples, a post-curing process was applied under 405 nm UV light for 4 min. All samples were conditioned at room temperature for 24 h prior to thermal and mechanical characterization.

A Design of Experiments (DoE) study was subsequently conducted to evaluate the process parameters of the selected optimal formulation. The DoE study was conducted as a “3-variable full-factorial” design, with a center point added to assess linearity. The total number of experiments was selected 2^3^ + 1 = 9. The DoE study was designed using Minitab^®^ Software (Minitab version 21), and the evaluation was also performed using the same program. The variables selected for the DoE study were layer thickness, exposure time, and post-curing time, while all other parameters were kept constant. The fixed process parameters used to determine the glass transition temperature during the formulation development stage, along with the minimum and maximum value ranges applied in the DoE study conducted to evaluate the process parameters of the selected optimal formulation, are summarized in Table 2. These values were established based on the experience gained during resin development and the fundamental operational constraints of the DLP device.

### 2.4. Characterization Methods

#### 2.4.1. Thermal and Thermomechanical Analysis

**Figure 2:**
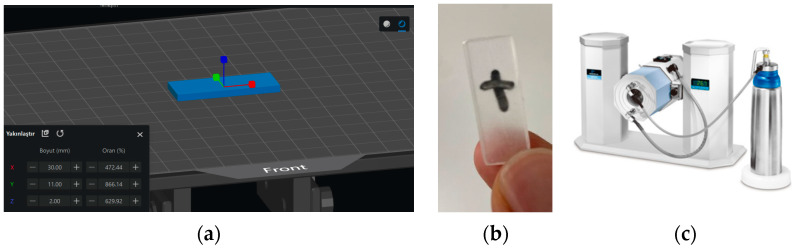
Test sample preparation: (**a**) DMA sample illustration for DLP 3D printing, (**b**) image of the printed sample, and (**c**) DMA device (PerkinElmer DMA 8000).

Dynamic mechanical analysis was performed using a PerkinElmer DMA 8000 analyzer (PerkinElmer, Inc., Waltham, MA, USA) to evaluate the thermal transitions and thermomechanical properties of samples printed with a photo-curable resin using a 3D printer. Rectangular samples (30 × 11 × 2 mm^3^) were tested in bending mode at a heating rate of 5 °C/min in the temperature range of 0 to 80 °C. The storage modulus (E′), loss modulus (E″), and tan δ were recorded. The T_g_ value was determined from the peak of the tan δ curve. The thermomechanical properties were assessed via storage and loss modulus. Images of the DMA sample illustration, the printed sample, and the DMA analyzer are presented in Figure 2.

#### 2.4.2. Shape Memory Analysis

**Figure 3:**
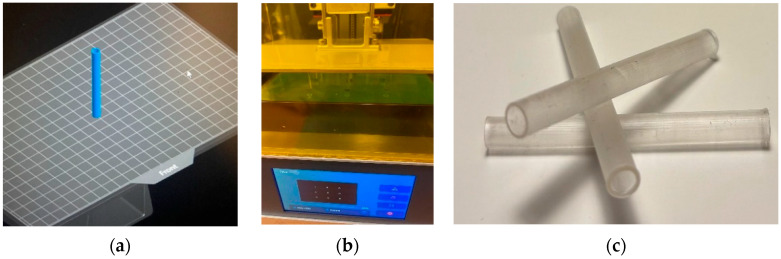
Shape memory test sample preparation: (**a**) conversion of the CAD data to STL format, (**b**) printing of the pipe shape sample on the DLP 3D Printer, and (**c**) image of the printed tubular samples.

The shape memory properties of UV-cured PUA resins were imparted by immersing them in pure water at controlled temperatures. Cylindrical hollow tubular samples (inner diameter: 8 mm, wall thickness: 1 mm, length: 100 mm) were prepared using DLP printing. The samples were first immersed in water above the glass transition temperature (T_g_ + 15 °C) and, after being held at this temperature for a period, were forced into a secondary shape. They were then immersed in water below the glass transition temperature (T_g_ − 15 °C), and the applied load was released. Subsequently, the samples were immersed in water at body temperature (37 ± 1 °C), and their return to their original shapes was observed. The preparation and printing process for the tubular sample used in the shape memory test, along with an image of the sample, are shown in Figure 3.

#### 2.4.3. Mechanical Analysis

Tensile tests were performed using an Instron 5982 electromechanical universal testing machine equipped with a 100 N load cell, in accordance with ISO 7198:2016. Type-IV specimens shaped like dog bones were prepared using DLP-based 3D printing and tested at a crosshead speed of 5 mm/min at room temperature. Tensile strength, elongation at break, and Young’s modulus were determined from the stress–strain curves. The breaking strength was calculated from the load at failure, while the breaking elongation was the percentage change in length at failure. However, since the Young’s modulus value is calculated in the literature based on blood vessel data, it was evaluated as the stress/strain ratio corresponding to a 30% elongation of the material. The Young’s modulus (E) was calculated using Equation (1), where σ_30%_ and ε_30%_ denote the stress and strain values at 30% elongation, respectively.

(1) $$E=\frac{\sigma_{30\%}}{\varepsilon_{30\%}}$$

**Figure 4:**
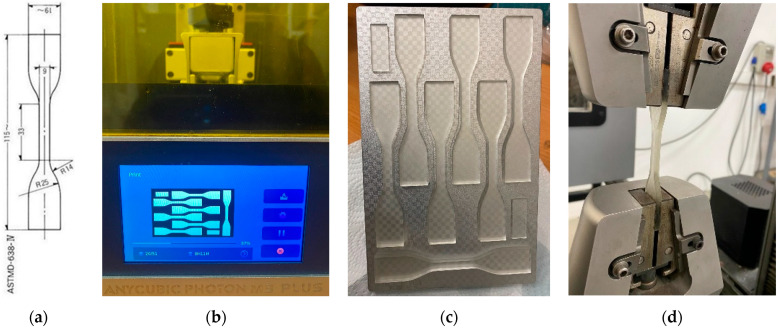
Mechanical test sample preparation: (**a**) technical drawing for ASTM D638 Type IV sample [52], (**b**) printing of the sample on the DLP 3D Printer, (**c**) printed samples on the table, (**d**) image of the sample during the tensile test.

To ensure repeatability, at least three samples (*n* ≥ 3) were tested for each resin formulation, and the data were evaluated based on the average of the two samples with the highest elongation values. The technical drawing of the tensile sample, the printing process, printed samples on the table, and the tensile test images are presented in Figure 4.

## 3. Results

The experimental results will be presented in two stages. The first stage involves selecting the appropriate resin formulation, while the second stage involves optimizing the process conditions.

### 3.1. Optimization for Resin Formulation

In the design of shape-memory polymers, the transition temperature between the glassy and rubbery phases is a critical parameter because, in thermally triggered systems, the shape-memory effect is primarily driven by this transition range [53]. The thermal transition’s peak is also defined as the T_g_ value, which is measurable by DMA and corresponds to the peak of the tan δ curve. For products designed for biomedical applications, the critical temperature is body temperature, 37 ± 1 °C [54], and therefore, initial studies were conducted to obtain a resin with a T_g_ in the range of 25–50 °C, which is close to this value. Table 3a presents the T_g_ values of the hard and soft resins obtained under the same process conditions for the two formulas presented. The use of hard polymers, such as high-chain UDMA, for Trial-1 resulted in harder products and a high T_g_ value (92.2 °C), whereas the use of soft structures, such as PEGDMA and LA, for Trial-2 resulted in softer polymers with a low T_g_ value (−4.2 °C). The values of these hard and soft polymers were evaluated using a linear line. The linear graph in Table 3b was used to determine the ratios in which Trial-1 and Trial-2 mixtures should be combined, with the aim of achieving a T_g_ value in the range of 25–50 °C. According to this graph, the compositions determined for Trial-3 (30% Trial-1 and 70% Trial-2) and Trial-4 (56.6% Trial-1 and 43.5% Trial-2) are presented in Table 3c.

**Figure 5:**
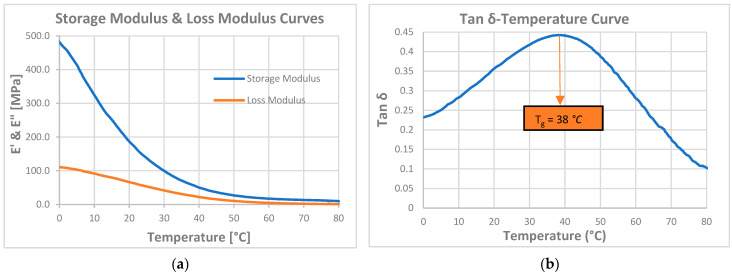
(**a**) Storage and loss modulus curves, and (**b**) Tan δ curve from the DMA for optimum resin formulation (Trial-3).

**Figure 6:**
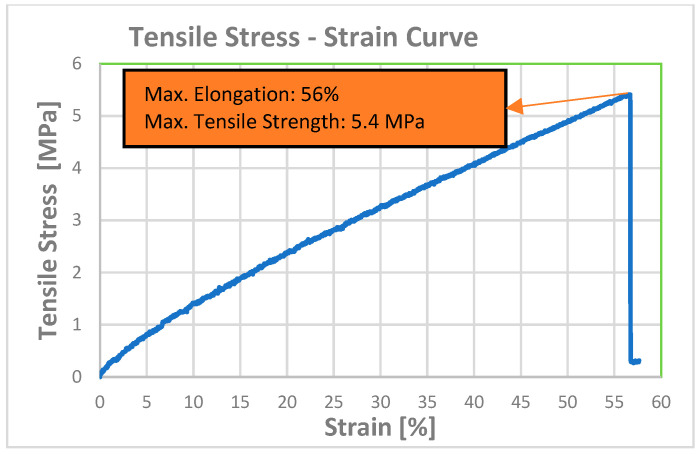
Stress–strain graph for optimum resin formulation (Trial-3) from the mechanical tensile test.

According to the DMA results obtained from the Trial-3 and Trial-4 experiments, T_g_ values of 38.0 °C and 51.0 °C were obtained, respectively. Based on these values, the linear evaluation was found inappropriate, indicating that the curing characteristics differed and that a non-linear approach should be used. However, since a value within the target T_g_ limits of 25–50 °C was obtained for the Trial-3 formulation, it was decided that this trial should be evaluated as a suitable resin formulation. Subsequently, the mechanical and thermomechanical properties along with the shape memory behavior of the optimized formulation (Trial-3) were evaluated. The storage modulus, loss modulus, and tan δ curves obtained from the DMA test are given in Figure 5. The tensile stress–strain curve obtained from the mechanical tensile test is given in Figure 6. Details of the procedure for the shape memory cycle, performed with a hot-water/cold-water test, are provided below.

The summary of values obtained from the thermomechanical and mechanical tests is given in Table 4.

According to the mechanical tensile test results, the elongation at break value obtained for Trial-3, the optimum formulation, is 53.5 ± 3.5%, the Young’s modulus is 10.9 ± 0.0 MPa, and the maximum tensile strength is 5.2 ± 0.2 MPa.

In the DMA test of Trial-3, which is performed to evaluate thermomechanical properties, the storage and loss modulus values obtained at human body temperature (37 °C) are 61 and 27 MPa, respectively.

The shape memory cycle procedure was applied to tubular samples printed by DLP as follows:

1. The initial stage of the cycle involves bringing the sample to thermal equilibrium. Here, the sample is allowed to reach a temperature of T_g_ + 15 °C.
2. The first step of the cycle involves high-strain deformation in a rubbery state, referred to as “pre-deformation” or “pre-stress.” This process involves the sample’s creep behavior at T_g_ + 15 °C under a constant load.
3. The second step is a “strain storage” process in which the material is cooled under “pre-stress constraint” so that ε_pre_ is preserved. This process determines the thermal behavior in which the temperature is lowered to T_g_ − 15 °C and the stress is changed while the strain is kept constant.
4. The third step is the “low-temperature unloading” process, defined as the removal of the strain constraint in the glassy state. This process describes the relaxation of stress on the sample at T_g_ − 15 °C. In this final stage of imparting shape memory properties, the material will have acquired a secondary shape at a temperature below T_g_.
5. The fourth step of the thermomechanical cycle, which involves reheating, is referred to as “free strain recovery” or “unconstrained recovery”; this means that there is no applied external stress and the induced strain is recovered freely. The material returns to its initial state. This process involves a thermal treatment that allows the shape memory material, which has been given a secondary shape, to return to its original shape by reheating to a temperature above T_g_ [54].

In the four-step process for shape memory cycling, with a T_g_ of 38 °C, the hot-water temperature is 53 °C, and the cold-water temperature is 23 °C. For this purpose, the above-mentioned formulation was printed as tubular samples on a DLP-based 3D printer under appropriate process conditions (layer thickness: 50 µm, exposure time: 8 s, post-curing time: 4 min). The entire process for these samples is described in Figure 5, numbered 1–5. In the first step, the sample was placed in water at 53 °C and left to wait (Stage-1, blue process). In the second step, the sample in water at 53 °C was bent to give it its secondary shape (Stage-2, orange process). In the third step, the secondary shape of the sample was fixed by removing it from 53 °C and quickly placing it in water at 23 °C (Stage-3, green). In the fourth step, the constraints forcing the secondary shape at 23 °C were removed, and after some recovery, the sample’s secondary shape was restored (Stage-4, red). In the final stage, the sample was returned to the initial temperature of 38 °C. It was observed that the material regained its initial shape when the water bath reached its starting temperature of 38 °C (Stage 5, purple).

**Figure 7:**
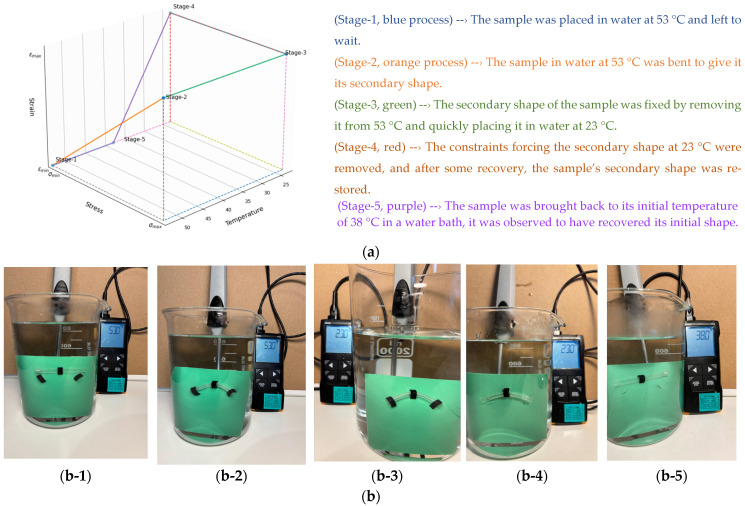
Shape memory cycle for tubular samples printed by DLP: (**a**) Schematic shape memory cycle and explanation (**b**) Five-step visual representation of the hot-water/cold-water test: (**b-1**) heating at 53 °C for a specific duration, (**b-2**) applying bending deformation at 53 °C to program the secondary shape, (**b-3**) cooling to 23 °C, (**b-4**) releasing the bending force at 23 °C, and (**b-5**) reheating to T_g_ = 38 °C to return to the original shape.

The processes involved in the shape memory cycle test are summarized in Figure 7, which includes 5 visuals. As seen in the last visual, at the end of the cycle, the bent material returned to a straight pipe shape similar to that in the first visual.

The mechanical performance of the optimized resin formulation was assessed by comparing its properties with those of natural vascular tissues and commercial vascular grafts. The Young’s modulus of Trial-3 was determined to be 10.9 ± 0.0 MPa, which falls within the circumferential modulus range (9–12 MPa) reported for human femoral arteries in the literature [9]. This correspondence suggests that the developed resin demonstrates compliance comparable to that of natural vascular tissue.

However, in terms of elongation at break, the measured value of 53.5 ± 3.5% remains below the reported range for human femoral arteries (63–76%), although it exceeds that of commonly used synthetic graft materials such as Dacron and Teflon, which typically exhibit elongation values in the range of 5–25% and are characterized by significantly lower ductility compared to native vessels [9]. These findings indicate that, while the optimized resin formulation achieves mechanical properties comparable to those of commercial synthetic grafts, its extensibility remains inferior to that of natural vascular tissues.

Therefore, to further enhance mechanical performance and more closely approximate the behavior of natural vessels, a process conditions optimization study based on Design of Experiments (DoE) was conducted.

### 3.2. Optimization for Process Conditions for Optimum Resin Formulation

The primary objective of optimizing process conditions is to improve the mechanical properties of the shape-memory-capable resin material. This will ensure that samples obtained from the resin have mechanical properties more similar to those of natural vessels. Since the mechanical properties of photo-curable resins depend on the curing stage and duration, the layer thickness, exposure time, and post-curing time parameters will be re-optimized to improve elongation at break, in particular, and to achieve improvements in other mechanical properties accordingly [55].

The use of DoE was planned to optimize these parameters, and this approach has previously been applied to such materials in the literature [47,56]. The design of the planned DoE study and the experimental results are presented in Table 5.

A linear multiple regression study was conducted for both elongation at break and Young’s modulus. The purpose of the regression study was to examine the effect of the variables on the process outcome and to develop a regression model that maximizes elongation. The results of the regression study predicting elongation at break are presented in Table A1.

Regression analyses were conducted to evaluate the effects of process parameters on the mechanical performance of the photocurable resin system, yielding highly statistically significant model fits for both elongation at break and Young’s modulus. The R^2^, adjusted R^2^, and predicted R^2^ values in the elongation-at-break model were 99.94%, 99.86%, and 99.60%, respectively, while in the Young’s modulus model, these values were 99.99%, 99.98%, and 99.96%, respectively. The high consistency of the adjusted R^2^ and predicted R^2^ values with the R^2^ value indicates that the models do not suffer from overfitting and possess strong generalization capabilities.

All statistical analyses were performed at a 95% confidence level (α = 0.05), and parameters with *p* < 0.05 were considered statistically significant. The ANOVA results indicate that the regression terms are statistically significant for both models (*p* < 0.001).

The layer thickness parameter has the most pronounced effect on elongation at break (F = 270.92; *p* < 0.001), while the statistical significance of the quadratic term indicates that the system exhibits non-linear behavior. The exposure time parameter showed borderline significance (*p* = 0.046), suggesting that the curing time may limit chain mobility.

When examining the regression model for Young’s modulus, the layer thickness and exposure time parameters have positive, statistically significant effects on the modulus (*p* < 0.001 and *p* = 0.009, respectively). The significance of the quadratic and interaction terms indicates that the system’s curing behavior is non-linear and that parameter interactions significantly affect mechanical performance. In particular, the positive coefficient of the triple interaction involving post-curing indicates that secondary curing increases cross-link density, thereby increasing rigidity.

When the results obtained are evaluated together, a typical inverse relationship between ductility and rigidity is observed in the system. Increasing the degree of cure and crosslink density reduces the elongation at break. This behavior is a mechanical result consistent with the restriction of chain mobility in photo-curable crosslinked polymer networks [57,58]. However, this behavior is not as simple as being explained solely by the increase in average crosslink density. In multi-component photopolymerizable resin systems, the oligomer structure, reactive diluent ratio, and combinations of photo-initiators significantly affect the heterogeneity of the network architecture. Factors such as differences in chain-length distribution, variations in local crosslink density, and microgel formation make the brittle-ductile transition a highly sensitive mechanical threshold [59]. Therefore, even small changes in process parameters can lead to significant differences in elongation at break.

The fact that layer thickness is the statistically dominant parameter can be attributed to the light-absorption spectrum and radical-generation kinetics of the TPO-1173 photo-initiator system used. When TPO’s high penetration ability is considered together with 1173’s radical-generating character near the surface, radical concentration gradients can form along the cure depth, depending on the layer thickness [60]. This situation leads to a non-homogeneous degree of network formation throughout the thickness and, consequently, to a mechanical response that is sensitive to process parameters. Therefore, the brittle-ductile transition observed in the system is shaped not only by crosslink density but also by complex interactions among light penetration, radical diffusion, and photopolymerization kinetics [59,61].

**Figure 8:**
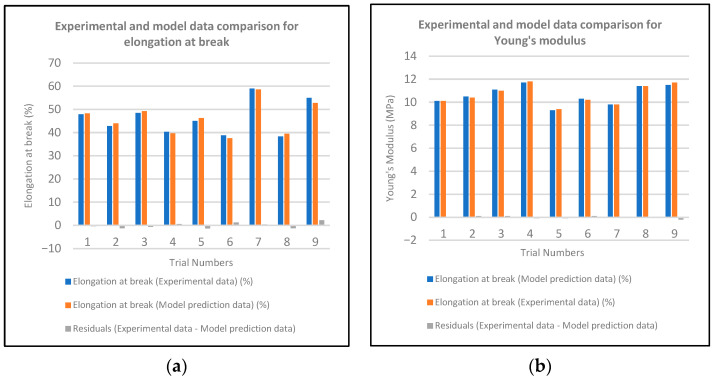
Comparison of experimental data with regression-predicted data for (**a**) elongation at break and (**b**) Young’s modulus.

Figure 8 presents a comparison of experimental data for elongation at break and Young’s modulus with regression-predicted data, showing the differences between them.

To validate model prediction performance, experimental and model predictions were compared, and residual analyses were performed. It was determined that elongation at break varied within a range of ±2.2, while Young’s Modulus varied within a range of ±0.2, showing no systematic trend. The balanced distribution of residual data in both positive and negative directions demonstrates the reliability of the regression models produced.

**Figure 9:**
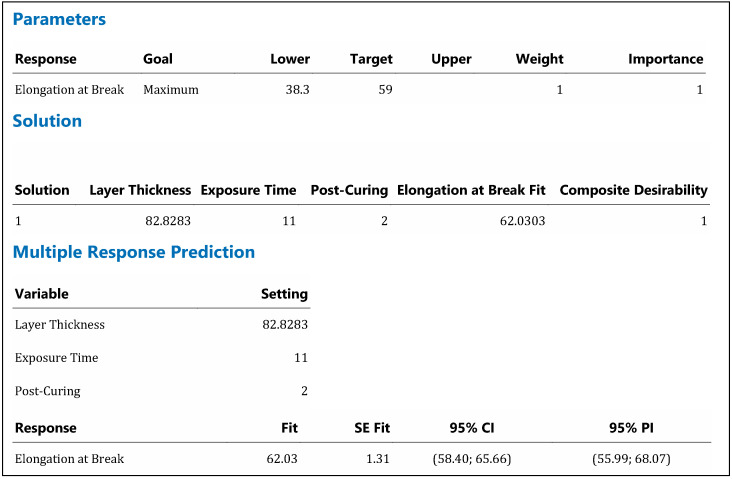
Prediction of process parameters that will yield maximum strain using regression analysis.

The data and graphs related to the regression studies are presented above. Multiple response optimization was performed using the desirability function approach. The optimal parameter combination, obtained to maximize elongation at break, is evaluated in Figure 9.

The optimal parameter combination, determined by maximizing elongation at break using the multi-response optimization and desirability function approach, was a layer thickness of 82.83 µm, an exposure time of 11 s, and a post-curing time of 2 min. Under these conditions, the model-predicted elongation at break is 62.03, falling within the 95% confidence interval (58.40–65.66) and the 95% prediction interval (55.99–68.07).

The optimum point, characterized by a high layer thickness and a medium exposure time, indicates a balanced cross-linking structure between network formation kinetics and chain mobility. This situation shows that the system operates in a mechanical regime close to the brittle-ductile transition threshold, but with ductile behavior more dominant.

**Figure 10:**
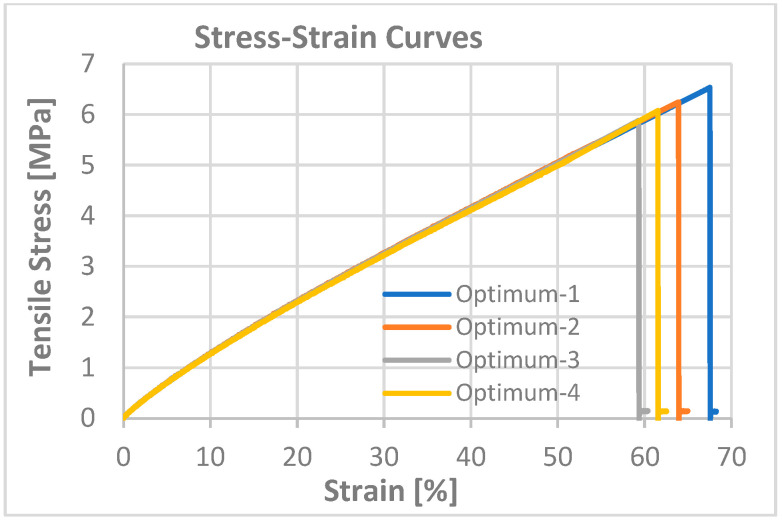
Stress–strain curve obtained from tensile test for optimum process formulation.

The results of the mechanical tensile test for the production to which the estimated process parameters from the regression analysis were applied are summarized in Table 6. The stress–strain curve is also presented graphically in Figure 10.

**Figure 11:**
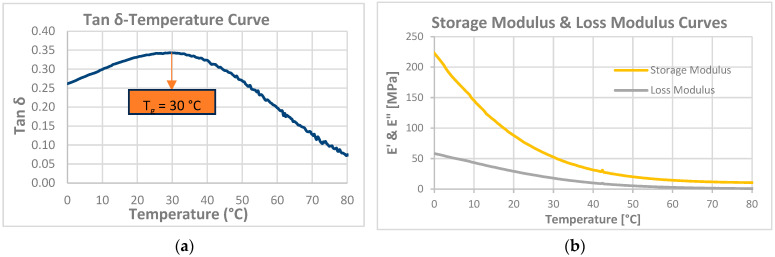
DMA test results for optimum process formulation, (**a**) tan δ curve, (**b**) storage modulus and loss modulus curves.

The results of the DMA test to assess the thermomechanical properties after the process optimization are summarized in Figure 11.

The optimum process parameters determined using the desirability function approach (82.83 µm layer thickness, 11 s exposure time, and 2 min post-curing time) have been experimentally validated. As a result of four repeated mechanical tests conducted under these conditions, elongation at break was determined to be 64.0 ± 3.4%. The obtained average value falls within the estimated value of 62.03% and the 95% confidence interval (58.40–65.66), confirming the high prediction accuracy of the regression model.

The Young’s modulus was measured at 10.9 ± 0.1 MPa, indicating high repeatability in mechanical performance under the curing conditions. The maximum tensile strength was 6.2 ± 0.3 MPa, and the macroscopic integrity of the network structure was maintained.

The DMA determined the T_g_ value to be approximately 30 °C. This value falls within the target range of 37 ± 10 °C for biomedical applications, indicating that the system may exhibit phase-transition-sensitive behavior near body temperature and that this point may correspond to a glassy-rubbery transition, demonstrating shape-memory properties. The obtained T_g_ value confirms that the optimized conditions create a balanced network between chain mobility and cross-link density.

As in formulation optimization, the mechanical properties obtained in the process parameter optimization study were evaluated by comparison with those of natural vascular tissues. The Young’s modulus value measured under optimal conditions, 10.9 ± 0.1 MPa, is consistent with the modulus range reported in the literature for human femoral arteries (9–12 MPa). This indicates that the developed system exhibits mechanical behavior compatible with that of natural arterial tissue.

When evaluated in terms of elongation at break, the obtained value of 64.0 ± 3.4% corresponds to the elongation range of the human femoral artery (63–76%). Considering the higher elongation values reported for the internal mammary artery and, in particular, the saphenous vein, the developed system does not possess a deformation capacity as high as that of venous tissue, but it falls within the arterial tissue range.

DMA results revealed that the storage modulus at body temperature (37 °C) decreased from 61 MPa to 37 MPa following process optimization. This reduction can be attributed to both the decrease in crosslink density associated with the optimized process parameters and the shift in T_g_ from 38 °C to 30 °C, which places the optimized material in the rubbery plateau at body temperature, in contrast to the pre-optimized formulation, which remained near its glassy-to-rubbery transition at 37 °C. Concurrently, the elongation at break measured by tensile testing increased from 53.5 ± 3.5% to 64.0 ± 3.4%, confirming the transition toward a more ductile mechanical response. Taken together, these findings indicate that process optimization reduced material stiffness and enhanced ductility, rendering the resin system more mechanically compatible with the requirements of vascular graft applications. This mechanical compatibility offers a potential advantage, potentially contributing to a more homogeneous stress distribution under pulsatile blood flow.

Overall, the simultaneous optimization of the resin formulation and process parameters has achieved the targeted performance in both shape memory behavior and biomechanical compliance by balancing the network architecture, crosslink density, and chain mobility.

## 4. Discussion

In this study, both the formulation and process parameters of a photopolymerizable multi-component resin system designed for DLP-based 4DP were systematically optimized.

In the initial phase, the objective was to achieve a glass-to-rubber phase transition temperature within the 25–50 °C range, which is critical for vascular graft applications. The Trial-3 formulation, with a T_g_ of 38 °C, was identified as a suitable resin formulation, indicating that shape memory behavior can be triggered at physiological temperatures. The shape memory performance of the Trial-3 formulation was qualitatively demonstrated via a hot/cold water cycling test. Upon heating the specimen above T_g_ in a water bath, the sample—previously deformed into a secondary shape—was observed to recover its original form, confirming thermally triggered shape recovery. It should be noted that in the developed system, shape memory activation does not occur via a binary switch but rather across a continuous glass-to-rubber transition range. In minimally invasive vascular applications, the material would be implanted in its compressed, temporary form at room temperature and would gradually transition toward its permanent shape as it equilibrates to body temperature. This gradual, passively driven activation is considered an inherent advantage for controlled clinical deployment. From a 4DP perspective, this thermally triggered, physiologically driven shape recovery represents the defining functional principle of the developed vascular graft: a DLP-fabricated construct that is 3D-printed in its permanent tubular geometry, programmed into a compact temporary form, and autonomously actuated by body temperature upon implantation—thereby fulfilling the core promise of 4D printing in a clinically relevant context.

Although the mechanical properties of Trial-3—the optimal formulation identified in the initial phase—are superior to those of commercially used synthetic graft materials such as Dacron and Teflon, the elongation at break remained comparatively low relative to the mechanical properties of natural vascular tissues reported in the literature [9]. Accordingly, process optimization was conducted in the second phase of the study with the dual objectives of shifting the T_g_ further below body temperature—thereby reducing the likelihood of requiring additional external heating during implantation—and improving the material’s mechanical properties toward those of natural vessels. In this phase, layer thickness, exposure time, and post-curing time were optimized using a Design of Experiments (DoE) approach. The developed regression models demonstrated high statistical fit (R^2^ > 99%) and reliably captured the influence of process parameters on mechanical performance. The identification of layer thickness as the dominant variable reflects the crosslinking behavior governed by the photo-initiator system’s light penetration depth and radical production kinetics. The optimal process conditions determined via the desirability function approach (layer thickness: 82.83 µm, exposure time: 11 s, post-curing time: 2 min) were subsequently validated experimentally. A pronounced effect of process optimization on the material’s thermomechanical response was observed: the storage modulus at body temperature (37 °C), as determined by DMA, decreased from 61 MPa to 37 MPa. This reduction is attributed to both the change in crosslink density associated with the optimized process parameters and the downward shift in T_g_ from 38 °C to 30 °C, which positions the optimized material within the rubbery plateau at body temperature. Since the T_g_ of the optimized resin is below body temperature, the material can spontaneously recover its original form under physiological conditions following implantation, potentially eliminating the need for external heating during the procedure. Concurrently, the elongation at break increased from 53.5 ± 3.5% to 64.0 ± 3.4% in tensile testing, confirming a transition toward a more ductile mechanical response. Collectively, these findings indicate that process optimization reduces material stiffness and enhances ductility, thereby rendering the resin system more mechanically compatible with the requirements of vascular graft applications.

The mechanical properties obtained in this study were benchmarked against values reported in the literature for natural vascular tissues and alternative polymer systems. The Young’s modulus of the optimized formulation (10.9 ± 0.1 MPa) falls within the circumferential modulus range reported for human femoral arteries (9–12 MPa), indicating a high degree of mechanical compatibility with native arterial tissue. The elongation at break of 64.0 ± 3.4% corresponds to the lower bound of the reported range for circumferential elongation in femoral arteries (63–76%), indicating that the developed system approaches the deformation capacity of arterial tissue. In contrast, commercially available synthetic graft materials such as Dacron (PET) and Teflon (PTFE) typically exhibit elongation at break values of 5–25% and Young’s moduli that are considerably higher than those of natural vascular tissues, which can result in compliance mismatch at anastomosis sites [9]. The PUA system developed in this study offers substantially greater mechanical compatibility than these conventional materials, which represents a critical advantage in mitigating intimal hyperplasia associated with compliance mismatch.

Unlike shape memory systems that rely on direct heating or the incorporation of nanofillers for athermal actuation, the DLP-processable polyurethane acrylate resin developed in this study achieves a T_g_ of approximately 30 °C through process optimization alone, enabling spontaneous shape recovery at physiological body temperature without external stimuli or composite modification. This intrinsic thermal responsiveness makes the material suited for 4D-printed vascular graft applications, where minimally invasive deployment and autonomous shape recovery under physiological conditions are critical design requirements.

The results demonstrate that the interplay between network architecture, crosslink density, and chain mobility in photopolymerizable multi-component resin systems is decisive for both mechanical performance and shape memory behavior. By providing a mechanical profile closer to that of natural vascular tissue and a thermally triggered phase transition at physiological temperatures, the optimized system emerges as a promising candidate for biomedical applications. In the broader context of 4D printing, the developed PUA-based resin system suggests that DLP technology holds potential not only as a geometric fabrication tool but also as a platform for developing stimuli-responsive vascular constructs capable of time-dependent functional adaptation—an approach that may contribute to the evolution of conventional static graft design toward more dynamic implant concepts.

Future studies should focus on the quantitative characterization of shape memory mechanics, long-term mechanical stability, fatigue behavior, biocompatibility assessment, and performance evaluation under hemodynamic conditions.

## 5. Conclusions

In this study, a photopolymerizable polyurethane-acrylate (PUA) resin system was developed and optimized for DLP-based manufacturing with potential vascular graft applications. The formulation was tailored to achieve a glass transition temperature within the physiological range, enabling thermally triggered shape memory behavior. Mechanical and thermomechanical results demonstrated that the optimized material exhibits properties comparable to soft vascular tissues, with improved compliance relative to conventional synthetic grafts. The material also showed effective shape fixation and recovery upon thermal activation.

Overall, the results establish a clear relationship between resin composition, network structure, and thermomechanical performance, providing a practical approach for tuning DLP-processable shape-memory polymers. The developed system shows strong potential for 4D printing of vascular structures.

## Figures and Tables

**Table 1:** Percentage composition of the initial resin formulation.

Materials	UM-2200C1	UDMA-UM3200	TEGDMA	IBOA	TMPTA	TPO	1173
(%)	38.0	38.0	17.0	3.0	3.0	0.5	0.5

**Table 2:** Fixed process parameters used in the formulation development stage and variable parameter ranges applied in the DoE study for the selected optimal formulation.

Process Parameters	Layer Thickness (µm)	Exposure Time (s)	Post-Curing Time (min)
Fixed process parameters for optimization of resin formulation	50	7	4
Lower and upper limits for DoE study	50–100	5–11	2–10

**Table 3:** Evaluation of the optimal resin formulation: (a) Unit formula, process parameters, and T_g_ value produced from DMA for Trial-1 and Trial-2. (b) Graph showing the T_g_ results obtained from Trial-1 and Trial-2 evaluated using a linear approach. (c) Unit formula, process parameters, and T_g_ value produced from DMA for Trial-3 and Trial-4.

(a) Unit formula, process parameters, and T_g_ value produced from DMA for Trial-1 and Trial-2				
Materials	Trial-1 (%)		Trial-2 (%)	
UM-2200C1	38.00		30.00	
UDMA-UM3200	38.00		0.00	
PEGDMA	0.00		25.00	
TEGDMA	17.00		3.00	
IBOA	3.00		10.00	
LA	0.00		25.00	
TMPTA	3.00		2.00	
TPO	0.50		3.00	
1173	0.50		2.00	
TOTAL	100.00		100.00	
Layer thickness (µm)	50			
Exposure time (s)	7			
Post-curing time (s)	4			
DMA (T_g_ value) °C	92.2		−4.2	
(b) The T_g_ results obtained from Trial-1 and Trial-2 evaluated using a linear approach				
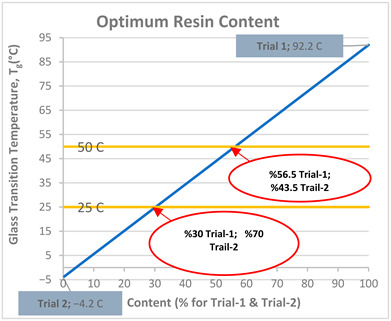				
(c) Unit formula, process parameters, and T_g_ value produced from DMA for Trial-3 and Trial-4				
Contribution ratios for Trial 3 and Trial 4	Trial-1 Ratio	Trial-2 Ratio	Trial-1 Ratio	Trial-2 Ratio
	%30.0	%70.0	%56.5	%43.5
Materials	Trial-3 (%)		Trial-4 (%)	
UM-2200C1	32.40		34.50	
UDMA-UM3200	11.40		21.50	
PEG600DMA	17.50		10.90	
TEGDMA	7.20		10.90	
IBOA	7.90		6.00	
LA	17.50		10.90	
TMPTA	2.30		2.60	
TPO	2.25		1.60	
1173	1.55		1.10	
TOTAL	100.00		100.00	
Layer thickness (µm)	50			
Exposure time (s)	7			
Post-curing time (s)	4			
DMA (T_g_ value) °C	38.0		51.0	

**Table 4:** (a) DMA test results, and (b) mechanical tensile test results for Trial-3, which has the optimum resin formulation.

(a) DMA Test Results	
Storage Modulus at 37 °C (MPa)	61.0
Loss Modulus at 37 °C (MPa)	27.0
T_g_ (°C)	38.0
(b) Mechanical Tensile Test Results	
Elongation at break (%)	53.5 ± 3.5
Young’s Modulus (%30) (MPa)	10.9 ± 0.0
Max. Tensile Strength (MPa)	5.2 ± 0.2

**Table 5:** DoE design, process parameters, and related mechanical analysis results.

DoE Trials	Process Parameters			Experimental Results		
	Layer Thickness (µm)	Exposure Time (s)	Post-Curing Time (s)	Elongation at Break (%)	Young’s Modulus (MPa)	Tensile Strength (MPa)
1	50	5	2	47.9 ± 7.6	10.1 ± 0.1	4.3 ± 0.7
2	50	5	10	42.8 ± 7.7	10.5 ± 0.3	4.1 ± 0.8
3	50	11	2	48.5 ± 5.9	11.1 ± 0.3	4.8 ± 0.4
4	50	11	10	40.3 ± 10.8	11.7 ± 0.3	4.4 ± 0.9
5	100	5	2	45.0 ± 0.7	9.3 ± 0.2	3.5 ± 0.0
6	100	5	10	38.8 ± 0.9	10.3 ± 0.0	4.0 ± 0.1
7	100	11	2	59.0 ± 8.6	9.8 ± 0.4	5.0 ± 0.8
8	100	11	10	38.3 ± 2.4	11.4 ± 0.1	4.2 ± 0.2
9	75	8	6	55.0 ± 3.6	11.5 ± 0.2	5.7 ± 0.5

**Table 6:** Individual results from mechanical tensile testing and DMA were conducted under optimal process conditions.

Optimum Process Parameters			Results		
Layer Thickness (µm)	Exposure Time (s)	Post Curing Time (min)	Mechanical Tensile Test		
			Elongation at Break (%)	Young’s Modulus (MPa)	Max. Tensile Strength (MPa)
82,83	11	2	68.2	10.8	6.5
			65.0	10.9	6.3
			60.4	10.9	5.9
			62.5	10.8	6.1
Average ± Standard Deviation			64.0 ± 3.4	10.9 ± 0.1	6.2 ± 0.3

## Data Availability

The original contributions presented in this study are included in the article. Further inquiries can be directed to the corresponding authors.

## Abbreviations

The following abbreviations are used in this manuscript:

DLP	Digital Light Processing
3DP	Three-dimensional Printing
4DP	Four-dimensional Printing
T_g_	Glass Transition Temperature
DMA	Dynamic Mechanical Analysis
SMP	Shape Memory Polymer
PLMC	Poly(d,l-lactide-co-trimethylene carbonate)
NIR	Near infrared
VP	Vat polymerization
SLA	Stereolithography
DMD	Digital Micromirror Device
DoE	Design of Experiment
ANOVA	Analysis of Variance
TIPS	Thermally Induced Phase Separation
PET	Polyethylene terephthalate
PTFE	Polytetrafluoroethylene
PUA	Polyurethane Acrylate
PU	Polyurethane
PCL	Poly-caprolactone
PLA	Polylactic acid
PGA	Polyglycolic acid
UM	Urethane methacrylate
UDMA	Urethane dimethacrylate
PEGDMA	Poly (ethylene glycol) dimethacrylate
TEGDMA	Triethylene glycol dimethacrylate
LA	Lauryl acrylate
IBOA	Isobornyl acrylate
TMPTA	Trimethylolpropane triacrylate
TPO	Phosphine oxide-based photo-initiator
1173	α-hydroxybenzophenone derivative photo-initiator

## Appendix A

**Table A1:** Regression analysis data for (a) elongation at break and (b) Young’s modulus.

(a) Regression analysis data for elongation at break					
Regression Equation					
Elongation at Break = 1.5420 Layer Thickness − 1.751 Exposure Time − 0.01182 Layer Thickness * Layer Thickness + 0.04238 Layer Thickness * Exposure Time − 0.002168 Layer Thickness * Exposure Time * Post-Curing					
Model Summary					
S		R-sq	R-sq (adj)	R-sq (pred)	
1.73651		99.94%	99.86%	99.60%	
Analysis of Variance					
Source	DF	Adj SS	Adj MS	F-Value	*p*-Value
Regression	5	19,593.9	3918.77	1299.55	0.000
Layer Thickness	1	816.9	816.94	270.92	0.000
Exposure Time	1	24.7	24.67	8.18	0.046
Layer Thickness * Layer Thickness	1	403.2	403.20	133.71	0.000
Layer Thickness * Exposure Time	1	86.7	86.67	28.74	0.006
Layer Thickness * Exposure Time * Post-Curing	1	274.5	274.53	91.04	0.001
Error	4	12.1	3.02		
Total	9	19,605.9			
Coefficients					
Term	Coef	SE Coef	T-Value	*p*-Value	VIF
Layer Thickness	1.5420	0.0937	16.46	0.000	161.90
Exposure Time	−1.751	0.612	−2.86	0.046	80.49
Layer Thickness * Layer Thickness	−0.01182	0.00102	−11.56	0.000	158.26
Layer Thickness * Exposure Time	0.04238	0.00791	5.36	0.006	83.11
Layer Thickness * Exposure Time * Post-Curing	−0.002168	0.000227	−9.54	0.001	3.47
(b) Regression analysis data for Young’s modulus					
Regression Equation					
Young’s Modulus = 0.27937 Layer Thickness + 0.2575 Exposure Time − 0.001878 Layer Thickness * Layer Thickness − 0.002396 Layer Thickness * Exposure Time + 0.000184 Layer Thickness * Exposure Time * Post-Curing					
Model Summary					
S		R-sq	R-sq(adj)	R-sq(pred)	
0.156088		99.99%	99.98%	99.96%	
Analysis of Variance					
Source	DF	Adj SS	Adj MS	F-Value	*p*-Value
Regression	5	1023.09	204.619	8398.57	0.000
Layer Thickness	1	26.82	26.816	1100.64	0.000
Exposure Time	1	0.53	0.534	21.92	0.009
Layer Thickness * Layer Thickness	1	10.18	10.178	417.75	0.000
Layer Thickness * Exposure Time	1	0.28	0.277	11.37	0.028
Layer Thickness * Exposure Time * Post-Curing	1	1.98	1.982	81.37	0.001
Error	4	0.10	0.024		
Total	9	1023.19			
Coefficients					
Term	Coef	SE Coef	T-Value	*p*-Value	VIF
Layer Thickness	0.27937	0.00842	33.18	0.000	161.90
Exposure Time	0.2575	0.0550	4.68	0.009	80.49
Layer Thickness * Layer Thickness	−0.001878	0.000092	−20.44	0.000	158.26
Layer Thickness * Exposure Time	−0.002396	0.000711	−3.37	0.028	83.11
Layer Thickness * Exposure Time * Post-Curing	0.000184	0.000020	9.02	0.001	3.47
